# Biosynthetic Strategies for Macrocyclic Peptides

**DOI:** 10.3390/molecules26113338

**Published:** 2021-06-01

**Authors:** Wei Wang, S. Cyrus Khojasteh, Dian Su

**Affiliations:** Drug Metabolism and Disposition, Genentech, 1 DNA Way, South San Francisco, CA 94080, USA; wang.wei@gene.com (W.W.); khojasteh.cyrus@gene.com (S.C.K.)

**Keywords:** macrocyclic peptides, bicyclic peptides, biosynthesis, ribosomal synthesis, chemoenzymatic strategy, library screening, in vitro display

## Abstract

Macrocyclic peptides are predominantly peptide structures bearing one or more rings and spanning multiple amino acid residues. Macrocyclization has become a common approach for improving the pharmacological properties and bioactivity of peptides. A variety of ribosomal-derived and non-ribosomal synthesized cyclization approaches have been established. The biosynthesis of backbone macrocyclic peptides using seven new emerging methodologies will be discussed with regard to the features and strengths of each platform rather than medicinal chemistry tools. The mRNA display variant, known as the random nonstandard peptide integrated discovery (RaPID) platform, utilizes flexible in vitro translation (FIT) to access macrocyclic peptides containing nonproteinogenic amino acids (NAAs). As a new discovery approach, the ribosomally synthesized and post-translationally modified peptides (RiPPs) method involves the combination of ribosomal synthesis and the phage screening platform together with macrocyclization chemistries to generate libraries of macrocyclic peptides. Meanwhile, the split-intein circular ligation of peptides and proteins (SICLOPPS) approach relies on the in vivo production of macrocyclic peptides. In vitro and in vivo peptide library screening is discussed as an advanced strategy for cyclic peptide selection. Specifically, biosynthetic bicyclic peptides are highlighted as versatile and attractive modalities. Bicyclic peptides represent another type of promising therapeutics that allow for building blocks with a heterotrimeric conjugate to address intractable challenges and enable multimer complexes via linkers. Additionally, we discuss the cell-free chemoenzymatic synthesis of macrocyclic peptides with a non-ribosomal catalase known as the non-ribosomal synthetase (NRPS) and chemo-enzymatic approach, with recombinant thioesterase (TE) domains. Novel insights into the use of peptide library tools, activity-based two-hybrid screening, structure diversification, inclusion of NAAs, combinatorial libraries, expanding the toolbox for macrocyclic peptides, bicyclic peptides, chemoenzymatic strategies, and future perspectives are presented. This review highlights the broad spectrum of strategy classes, novel platforms, structure diversity, chemical space, and functionalities of macrocyclic peptides enabled by emerging biosynthetic platforms to achieve bioactivity and for therapeutic purposes.

## 1. Introduction

Macrocyclic peptides, including monocyclic and bicyclic peptides, are privileged molecular modalities which can be used for diagnosis (e.g., biosensors, glucose sensors), disease treatment (e.g., antimicrobial, cancer therapy), and drug delivery [1,2]. Although peptide therapeutics have greater potency compared to the small-molecule therapies, they often suffer reduced bioavailability due to their limited permeability and metabolic stability, which in turn decreases their clinical efficacy [3]. Macrocyclization, as one feature shared by structurally diverse molecules, generally improves several pharmacological features of a peptide, revealing the potential as a novel method for improving bioactivity [4]. Macrocyclic peptides have outstanding properties as compared to the linear form, with increased specificity and affinity toward the target protein and enhanced proteolytic resistance, therefore improving their specific potencies in vivo [5,6,7,8,9]. The design of peptide macrocyclization methods has become an important part of strategies.

Various macrocyclization reactions have been designed over the years, utilizing different mechanisms such as backbone cyclization and sidechain to sidechain cyclization [10,11,12], where backbone cyclization presents the most conformational constraint. Commonly, macrocyclic peptides are produced by non-ribosomal peptide synthetases [13,14,15,16] or ribosome-derived peptides by enzymatic posttranslational modifications [17,18,19]. Considering the chemical variety and stereochemical complexity, special emphasis on the selective transformation of polycyclic compounds arises for methods using enzymes and microbial whole cells. The use of microorganisms allows for obtaining enantiomerically pure compounds via one-stage synthesis [20].

**Figure 1:**
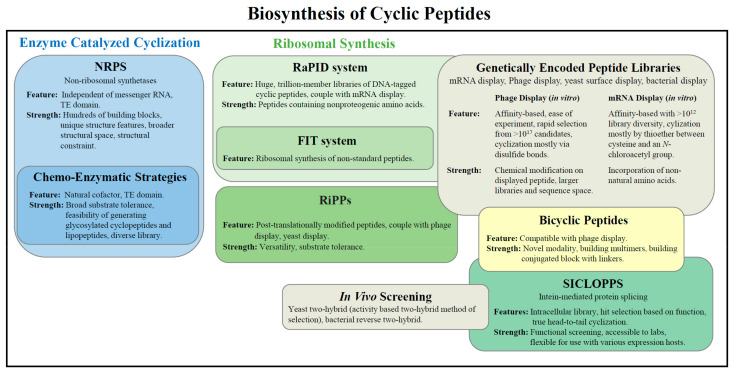
An overview of the platforms discussed in this review. The biosynthesis of bicyclic peptides is highlighted in yellow (FIT: flexible in vitro translation; RaPID: random nonstandard peptides integrated discovery; RiPPs: ribosomally synthesized and post-translationally modified peptides; SICLOPPS: split-intein circular ligation of peptides and proteins; NRPS: non-ribosomal synthetase; TE domain: thioesterase domain).

In particular, the use of genetically encoded peptide libraries constitutes an attractive strategy to generate large collections of these molecules, which have been amenable to screening through display platforms to accelerate the discovery of cyclopeptide binders [21,22,23,24]. For instance, in vitro translation of cyclic peptides [25,26,27] or cyclization of mRNA- or phage-displayed peptides via chemical [28,29,30,31] or enzymatic processes [20,32], have been successfully applied for this purpose, resulting in the identification of cyclic peptides capable of interacting with a variety of targets. The respective methods disclosed in this review (Figure 1) will provide new opportunities to prepare collections of macrocyclic peptides and bicyclic peptides, as well as composed libraries with a variety sequence levels and chemical diversity, with a focus on biosynthesis. More importantly, features, advantages as well as insights into peptide cyclization platforms and perspectives for future development will be discussed.

## 2. Biosynthetic Strategies

### 2.1. Flexible In Vitro Translation (FIT)

**Figure 2:**
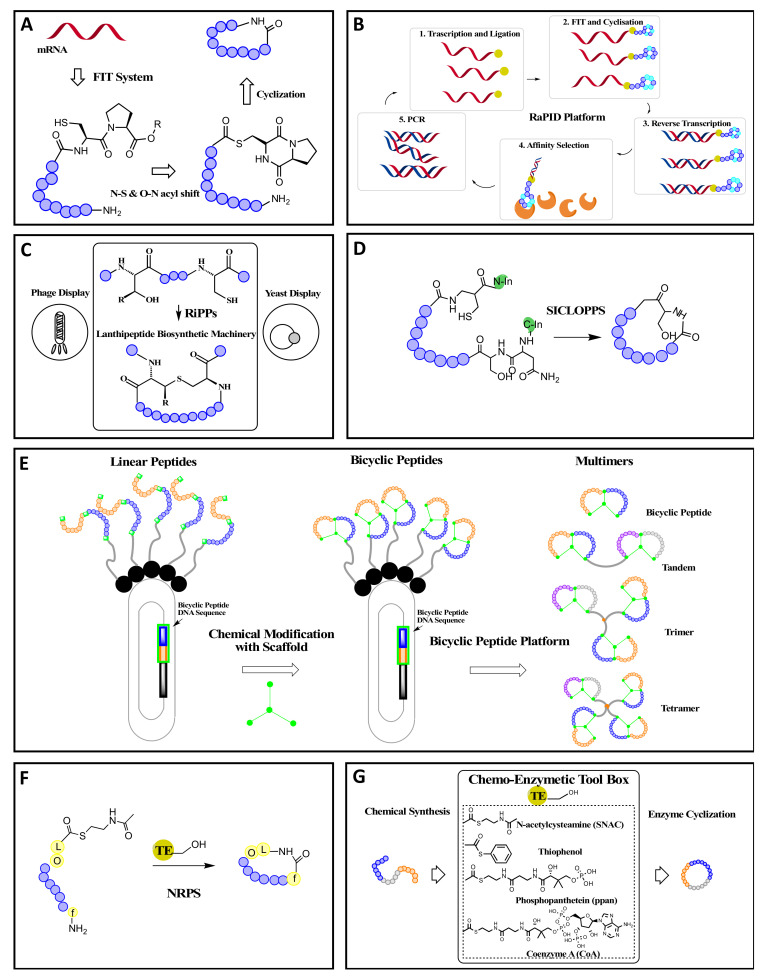
Illustrations of cyclic peptide synthesis platforms. (**A**) FIT (adapted from [3] with permission from The Royal Society of Chemistry); (**B**) RaPID (adapted from [37] with permission from The Royal Society of Chemistry); (**C**) RiPPs (adapted from [20]); (**D**) SICLOPPS (adapted from [47]); (**E**) Bicyclic peptide synthesis based on phage display and chemical cyclization (adapted from [48] and Bicycle Therapeutics website https://www.bicycletherapeutics.com (accessed on 21 May 2021); (**F**) NRPS (adapted from [47]); and (**G**) Chemo-enzymetic synthesis (adapted from [13]). (FIT: flexible in vitro translation; RaPID: random nonstandard peptides integrated discovery; RiPPs: ribosomally synthesized and post-translationally modified peptides; SICLOPPS: split-intein circular ligation of peptides and proteins; NRPS: non-ribosomal synthetase).

The flexible in vitro translation (FIT) system comprises the integration of a reconstituted *E. coli* translation system and NAA-tRNAs prepared by flexizymes (artificially evolved ribozymes able to catalyze the aminoacylation of tRNA with a variety of non-proteinogenic amino acids (NAAs). FIT facilitates expression of various peptides containing NAAs from designed mRNA templates according to the newly designated genetic table by means of genetic code reprogramming (Figure 2A) [3,25,33,34,35]. Therefore, the advantage of the FIT system as a cyclization method is the incorporation of more NAAs as the backbone macrocyclic peptides [1,36]. However, the limitation of the use of this system alone is the lack of utilization of a theoretically full capacity for the library [3].

### 2.2. Random Nonstandard Peptides Integrated Discovery (RaPID)

mRNA display is a reliable methodology for mass peptide library screening (∼10^13^ members) and has been used for peptide drug discovery (Figure 2B) [21,22,37]. It is superior to other screening methodologies in terms of rapidness and peptide selection based on affinity potencies against the proteins of interest. The combination of the mRNA display and FIT [38] was named the “random nonstandard peptides integrated discovery” (RaPID) system, enabling the accessibility and mass screening of peptides containing NAAs [25] and cyclization compatible with mRNA display. In the RaPID system, in vitro translation is modified to use reprogrammed genetic codes to enable spontaneous peptide macrocyclization, including NAAs such as α-hydroxy acids, *N*-methyl-, D-, β-amino acids, and amino acids bearing nonstandard sidechains [27,33,39,40,41]. One cyclization technique utilizes the N-terminal installed chloroacetyl functionality to spontaneously cyclize with an internal cysteine under translation conditions, while other approaches for cyclization are also available [27]. The main feature of RaPID is the generation of huge, trillion-member libraries of DNA-tagged cyclic peptides [27]. The use of these libraries for target engagement, followed by DNA sequencing, enables selection of highly specific, tight-binding cyclic peptide sequences [27,42]. Furthermore, the advantages of RaPID include the use of diverse molecular topologies of macrocyclic peptides to generate a trillion unique members and enrichment or enhancement for low-abundance, high-affinity ligands [43].

As the first showcase of this system, Yamagishi et al. utilized it for selection of anti-E6AP macrocyclic *N*-methyl-peptides, where one of the abundant classes of selected peptides exhibited an inhibitory activity against E6AP-catalyzed polyubiquitination of target proteins such as p53 and peroxiredoxin 1 [25]. This work demonstrated the potential of the RaPID system for the discovery of a novel class of nonstandard peptides against previously non-druggable targets [25]. Another highly synthetic approach reported by Nawatha et al. integrated both the chemical synthesis of proteins and screening against trillion-member macrocyclic peptide libraries using RaPID, making post-translationally modified targets accessible for drug discovery [27]. The selected new cyclic peptides can bind tightly and specifically to K48-linked Ub chains, protect K48-linked Ub chains from deubiquitinating enzymes, and prevent proteasomal degradation of Ub-tagged proteins, opening up new opportunities for therapeutic interventions [27]. Other original cases utilizing RaPID have been used to discover inhibitors of cofactor-independent phosphoglycerate mutase (iPGM) [43] and isoform-selective Akt kinase inhibitors [44], as well as potent macrocyclic inhibitors of PD-1/PD-L1 interactions [45,46]. Overall, RaPID screening has been proven to be a powerful system for the discovery of bioactive macrocyclic peptides, and has been used to generate a number of high-affinity, highly selective binders.

### 2.3. Ribosomally Synthesized and Post-Translationally Modified Peptides (RiPPs)

Ribosomally synthesized and post-translationally modified peptides (RiPPs) are an emerging class of natural products with drug-like properties. RiPPs represent a particular platform with promising perspectives for engineering new functionalities using a direct link between a gene-encoded precursor peptide and the final macrocyclic compound [49]. To fully exploit the potential of RiPPs as drug candidates, Urban et al. reported a phage display-based tool for systematic engineering with examples of lanthipeptides, a subclass of RiPPs characterized by multiple thioether cycles that are enzymatically introduced in a regio- and stereospecific manner (Figure 2C) [20,32]. The phage display system was found to be suitable for generating lanthipeptide ligands for protein targets and could be adapted to other library designs [50]. Furthermore, the *C*-terminal display on pIII could be applicable for the engineering of other RiPP classes, and acts as a valuable source to identify therapeutic peptide alternatives [32]. Hetrick et al. demonstrated the successful display and randomization of both class I and II lanthipeptides with the versatility and potential of the RiPP display [20]. The lanthipeptide biosynthetic enzyme systems were demonstrated to be amenable for the display of lanthipeptides by *N*- or *C*-terminal phage display and yeast display, suggesting strong applications [20]. These blueprints for the display of RiPPs on either yeast or phages allow for the full exploration of the remarkable substrate tolerance of RiPP biosynthetic enzymes, with structure versatility compared to conventional methods.

### 2.4. Split-Intein Circular Ligation of Peptides and Proteins (SICLOPPS)

Despite significant contributions in the biosynthetic area, the above approaches have to date remained largely limited to the production of macrocyclic peptides in vitro [22,51]. Another novel discovery approach relying on the *in cellulo* production of macrocyclic peptides is the split-intein circular ligation of peptides and proteins (SICLOPPS) method [4]. For instance, Lennard et al. developed a cyclic peptide inhibitor of p6/UEV protein−protein interaction identified using SICLOPPS screening [52]. Intein-mediated protein splicing is a posttranslational processing of proteins in which an internal protein sequence is removed from a protein precursor, and its *N*- and *C*-terminal flanking fragments, named exteins, are ligated to each other [3,53,54]. The internal part of protein, named intein [55], possesses the catalytic activity necessary to carry out protein splicing, e.g., the cleavage of two amide bonds and the formation of a new amide bond [56]. In general, backbone cyclic peptides are generated via the circularization of an internal peptide sequence upon a trans splicing reaction involving flanking domains from the natural split intein DnaE [57]. SICLOPPS utilizes the natural process of intein spicing to generate macrocyclic peptides (Figure 2D) [47,57]. By placing a randomized sequence between a *C*-terminal and *N*-terminal intein domain, a backbone macrocyclic peptide library is generated as a byproduct of the intein splicing process [57]. The general method to produce macrocyclic peptides using intein chemistry involves a process in which an intein domain is arranged at the *C*-terminal region of the objective precursor peptide and an *N*-terminal Cys bearing a free amino group is formed by an appropriate sequence-specific peptidase such as methionine aminopeptidase [3]. The advantages of the SICLOPPS screening platform include easy accessibility with regard to laboratory capabilities [58], functional (rather than affinity-based) screening if coupled with activity-based two-hybrid selection, compatibility with different expression hosts such as *Escherichia coli*, yeast display [59,60,61,62] and human B cell display [63], true head-to-tail cyclization, and intracellular screening [24,64], all of which could significantly increase the number of bioactive hits during selection. Compared with other discovery platforms, limitations include the constraint of the tolerance of its host cell, challenges in system modifications and evolution, and limited accessibility of a single type of cyclic peptide topology [65].

### 2.5. Biological Synthesis of Bicyclic Peptides

Bicyclic peptides, which are widely distributed in nature and have increased proteolytic stability and conformational rigidity compared to monocyclic peptides, allow for a novel modality with improved parameters such as target binding affinity and selectivity [66]. Bicyclic peptides can be synthesized biologically via ribosomal synthesis and SICLOPPS [57,66]. For ribosome-derived synthesis, cyclization is converted by reaction with a small molecule scaffold such as tris(bromomethyl)benzene (TBMB) and 1,3,5-triacryoyl-1,3,5-triazinane (TATA) [67,68,69,70,71], phage display via the formation of two disulfide bonds [29], and mRNA display with reprogramming for incorporating of unnatural amino acids [66,72]. Alternatively, the SICLOPPS method integrated with the genetic incorporation of unnatural amino acids could produce natural product-like bicyclic peptides of varied ring sizes [73]. Recently, Upadhyaya et al. discovered BT7480 as a synthetic tumor-targeted CD137 agonist to induce anticancer immunity [48]. This is known as a tumor-targeted immune cell agonist (TICA) based on its constrained bicyclic peptides via the phage library display (Figure 2E) [48] (Bicycle Therapeutics website https://www.bicycletherapeutics.com, accessed on 21 May 2021). As a *Bicycle*^®^ TICA, BT7480 is a heterotrimeric conjugate with 1 Nectin-4 and 2 CD137 *Bicycles*^®^ via linkers [48]. Benefiting from high versatility for the multimerization and conjugation of the modular platform, bicyclic peptides could be implemented for assembling multimers including tandems, trimers, tetramers and drug conjugates, which could be applied as standalone therapeutics [48]. The unique bicyclic peptides screening platform based on phage display could also be used for selection of bicyclic peptides with amenable links to other molecular payloads such as cytotoxins or other bicyclic peptides, to create complex molecules with combinatorial pharmacology [48]. Taking drug conjugate as an example, a tripartite complex is formed via bicyclic peptide conjugates through: (1) bicyclic peptides binding to a specific tumor antigen; (2) a designed, selectively cleavable linker (only by enzymes within specific microenvironment); and (3) a payload (small molecule) [48,74]. The toxic payloads are delivered into solid tumors via the linker and coupling chemistry, with very limited payload exposure to minimize toxicity achieved by the ideal PK and rapid tumor penetration (Bicycle Therapeutics website https://www.bicycletherapeutics.com, accessed on 21 May 2021). More importantly, bicyclic peptides are promising therapeutics that present a novel and flexible platform of building blocks to address intractable challenges for oncology medicines and other therapeutic practice [48]. Future developments could include the integration of one of the peptide display techniques to allow for combinatorial libraries of natural product-like bicyclic peptide screening against targets.

### 2.6. Enzyme-Catalyzed Peptide Cyclization

#### 2.6.1. Non-Ribosomal Synthetase (NRPS)

Challenges for chemical synthetic macrocyclization could be concerned with the inclusion of the steric repulsion of ring residues, ensuring regio-chemistry as well as decreased yields. Furthermore, enzyme-catalyzed peptide cyclization has been exploited due to the cost-effectiveness and high chemo-selectivity of biocatalysts [75]. Non-ribosomal machinery has been discovered for peptide synthesis using multienzyme complexes as an assembly line to catalyze stepwise peptide cyclization. Common post-synthetic modifications by enzymes associated with the NRPS machinery include glycosylation and oxidative cross-linking [13], which are mostly accomplished by a thioesterase domain (TE domain, also referred to as a peptide cyclase) fused to the *C*-terminal module (Figure 2F) [14,15,47,76]. This reaction could generate a linear acid through hydrolysis or release a cyclic peptide by an intramolecular reaction with an internal nucleophile. For instance, hydrolytic release is observed for vancomycin with a backbone constrained by post-synthetic oxidative cross-linking reactions [77]. Second example is surfactin, for which the peptide backbone is constrained by the intramolecular nucleophilic attack of a hydroxyl group of the fatty acid moiety for a branched chain lipodepsipeptide [78]. As another example, an isolated TE demonstrated the capability of catalyzing the cyclization of linear peptides via a phosphopantetheine linker and building a cyclic peptide library derived from the antibiotic tyrocidine [14,15,75]. In fact, a variety of functional peptide products were reported via NRPS subunits, such as bleomycin (antitumor) and cyclosporin (antifungal) [2]. Furthermore, macrocyclization catalyzed by non-ribosomal TE domains shows several advantages, such as independence of messenger RNA, the diversification and rigidification by tailoring enzymes, structural constraints in resistance to proteolytic degradation, specialization for cyclization reactions, versatile structural and mechanistic peptide cyclases [47], the incorporation of NAAs, and the implementation of heterocyclic rings and fatty acids, which open the door of the structure space to tons of building blocks [47].

#### 2.6.2. Chemo-Enzymatic Strategies

Chemo-enzymatic strategies have been developed to combine chemical linear peptide synthesis with enzymatically catalyzed cyclization to reprogram existing non-ribosome-derived produced peptides, utilizing nature-developed stereo- and regioselective peptide cyclization enzymes (Figure 2G) [13]. This combination highlights the benefits of the easy synthesis of linear peptide sequences by established solid-phase peptide chemistry and selective and efficient enzymatic cyclization [79,80]. Furthermore, based on the diversity of natural cyclization strategies, chemoenzymatic approaches for the cross talk between biology and chemistry present a new source of diversative cyclic peptides with altered features and diversification. To establish translation between the language of chemistry and biology via chemical mimicking of the biological pathway, a link between natural and artificial systems compatible with both was proposed. For instance, a short-mimicked copy of a natural cofactor (such as phosphopantetheine (ppan) *N*-acetylcysteamine (SNAC)) was attached to the *C*-terminal end of a chemically synthesized linear peptide (such as tyrocidine peptide) [81]. For instance, SNAC substrates were reported to characterize the cyclization of gramicidin S and surfactin [78,82]. Importantly, further studies suggested the specificity of *C*- and *N*-terminal residues of the substrate recognized by the tyrocidine peptide cyclase, even with different substrate lengths, stereochemical features, and amino acid sequences, demonstrating flexibility and a great tolerance for the modification and replacement of residues within the peptide backbone [82]. Future research efforts could include the generation of custom-made catalysts for cyclization of a specific sequence, the use of excised TE-domains [80], and investigation of the efficiency of combinatorial cyclization using these enzymes [13,14,15]. The advantages of chemoenzymatic approaches include broad substrate tolerance, the feasibility of producing glycosylated cyclopeptides and lipopeptides, and the extension of carbohydrate complexity into peptides as a useful toolkit for a large cyclic library search [13].

## 3. Discussions and Perspectives

Macrocyclic peptides and bicyclic peptides represent a golden middle ground as they have a size between that of small molecules and biological ligands, have functional attributes including unique selectivity, versatility, and structural stability, and are promising alternatives to small-molecule and macromolecule scaffolds [1]. The biosynthesis of cyclic and bicyclic peptides has attracted particular attention, bearing promising advantages over traditional methods. Each of reviewed platforms boasts its own strengths and has its own features, as reviewed above. The highlighted insights were summarized with regard to their perspectives.

### 3.1. Integration with the Library

Although various chemical and biological approaches allow the synthesis of diverse backbone macrocyclic peptides, coupling with libraries compatible with high-throughput screening methods drastically escalates screen capacity and allows for the fast identification of desired bioactive peptides against targets. Genetically encoded libraries of cyclic peptides generated through various approaches such as phage display, mRNA display, and split-intein circular litigation are increasingly being applied for macrocyclic compounds, with their own advantages and disadvantages [83]. Phage displays benefit from large library sizes and the ease of experiments [84]. One strength of phage displays is the potential to generate much larger libraries and sequence spaces, providing broader explorations for studied domains [85]. These chemistries can also be used to generate bicyclic peptide libraries considering that bicyclic peptide phage libraries can further be generated by chemical cross-linking. Another attractive in vitro method for library production is the mRNA display, which allows for the insertion of non-natural amino acid residues and peptide cyclization [83]. The library construction methodologies and their screening innovatively promote synthesis efficiency. For instance, disulfide-bridged cyclic peptides are identified by phage displays as the first inhibitors of HIV integrin (IN) and the cellular cofactor lens epithelium-derived growth factor (LEDGF/p75) [86]. Additionally, another emerging library via yeast surface display has also been used for efficient isolation and characterization of cyclic peptides produced from combinatorial libraries, suggesting that the yeast surface display enables selectivity and affinity screening [87]. Compared to in vitro screening, *in cellulo* screening may be limited by library sizes; nevertheless, this approach has other benefits for successful candidates; for instance, peptides which are soluble and more resistant to proteolytic degradation, are more likely to be screened by the cell host [88,89]. Future work should focus on developing screening approaches that better relate the peptide selection to its function of inhibiting its target, the selectivity for the target rather than competitor proteins, and peptide property (such as bioavailability) optimization to provide good therapeutics [88].

### 3.2. Combinatorial Libraries

An interesting strategy is the combination of in vitro and *in cellulo* approaches in tandem to benefit from the advantages of large library size and desirable properties through in-cell selection [88]. In addition, combinational insights applied for various libraries will assist in the future trend of cyclic peptide screening. The yeast surface display in combination with phage display has been shown to be an approach for the quick identification of protein binders and the filtering of non-functional peptides [90]. For example, the yeast surface display was implemented to select peptides binding to wild-type IL-23, helping to envisage the phage display as a future strategy for drug leads [90].

### 3.3. Advantages of Two-Hybrid Screening

The intracellular environment provides the unique advantage of activity-based selection [64]. Reverse two-hybrid screening could be coupled with a high-throughput platform for candidates in vivo [1]. Only SICLOPPS is compatible with activity-based two-hybrid screening, which makes it more reliable for discovering bioactive hits, whereas the other methods are more prone to discovering high-affinity binders that lack biological activity.

### 3.4. Structural Diversification

Non-ribosome-derived peptides exhibit structural diversity in terms of D-configured residues, oxidation, methylation, halogenation, lipidation, heterocyclization, and macrocyclization [13]. This feature is reflected in a broad spectrum of biological activities of non-ribosome-derived peptides, including antibacterial, immunosuppressive, and antitumor properties. Acidic lipopeptide antibiotics have demonstrated the structure versatility of non-ribosomal derived peptides such as calcium-dependent antibiotics (CDAs), daptomycin, A54145, friulimicins, and amphomycins [91,92,93,94,95,96]. These attributes will allow for the design of novel and diversified classes of bioactive peptides.

### 3.5. Inclusion with NAAs

The chemical space of available peptides is expanding via the synthesis of peptides containing NAAs with the aim of adding more functionalities such as stability, or to form specific tertiary structures to discover peptide therapeutics [97,98]. Nowadays, macrocyclic peptide-compatible screening technologies can also be tolerated with the introduction of peptides containing NAAs. For instance, the RaPID approach benefits from the possibility of incorporating unnatural amino acids through the FIT [83]. Thus, a larger chemical space will be available for the bioactive peptide using synthetic and screening methods together, for example RaPID, RiPPs, and even chemoenzymatic synthesis [1].

### 3.6. Chemoenzymatic Combination

The combinatorial method could provide large libraries of macrocyclic peptides created with both natural and unnatural amino acids as well as building blocks, which can subsequently be screened for novel or improved bioactivity. Furthermore, alterations in the substrate specificity of TE-domains by directed protein evolution will increase the utility of these macrocyclization catalysts [13]. However, little is known about the chemoenzymatic potential of tailoring enzymes, which can contribute to the structural diversity and rigidity of non-ribosomal derived peptides, including their tolerance in vitro for their specified reactions. Furthermore, it could be questioned as to whether exercised TE-domain swapping is an applicable tool for the production of novel peptides in vivo [13].

### 3.7. Expanded Toolbox

Except for the established platforms, other biosynthetic methodologies for macrocyclic peptides have recently been developed in living cells via the spontaneous, post-translational cyclization of recombinant polypeptides to produce and screen macrocyclic peptide libraries *in cellulo* for new discoveries [99,100]. The overall philosophy of new methodologies needs to expand the “toolbox” in order to expand opportunities in many ways with: (1) the creation of structurally and functionally diverse libraries of peptide macrocycles; (2) compatibility with functional assays; (3) integration with high-throughput display platforms; (4) genetic build-in structural features; (5) efficiency to produce cyclics with various ring size and amino acid sequences; (6) predictability of regioselectivity; and (7) amenability to coupling with well-established display platforms or intracellular selections [65,99,100]. Iannuzelli et al. developed and characterized an expanded toolbox of unnatural amino acids suitable for directing the biosynthesis of thioether-linked macrocyclic peptides via a cysteine cross-linking reaction by means of electrophilic non-canonical amino acids in bacterial cells [100]. These new cyclization strategies benefited from several features: the functionality and efficiency of electrophilic unnatural amino acid-mediated cyclic peptide formation; the variability of inter-sidechain linkages; enabling 21 amino acid residues *in cellulo*; various ranges of cyclization from short to long; accessibility with respect to broader scaffolds; and variations in the unnatural amino acid modules for function modulation. Bionda et al. developed methodologies to guide the production of “natural product-like” macrocyclic peptides constrained by an intramolecular thioether bridge in bacterial cells via the combination of a chemo-selective reaction (between encoded cysteine and a cysteine reactive unnatural amino acid) via intein-catalyzed protein splicing, inspired by the biosynthetic logic of natural products [65].

### 3.8. Insights on Bicyclic Peptides

The breakthrough application of phage displays in bicyclic peptides opens the door for bicyclic peptides, multimers and drug conjugates. Although many aspects (such as antitumor efficacy, toxic effects, and PK) need to be addressed as bicyclic peptides classes move into clinical phases, library-based bicyclic peptide platforms enable a new path for a wide range of therapeutics. The biosynthetic properties of bicyclic peptides critically enhance the versatility and functionality of macrocyclic peptides in drug discovery. The conjugate concept of multimers and block building via linkers provides a novel chemical approach for delivering payloads and active antitumor response. Furthermore, future investigation could advance biosynthetic approaches and perspectives of bicyclic peptides to other drug modalities.

### 3.9. Future Perspectives

Regarding the methods discussed in this review in terms of their features and strengths, further elaboration and development will embrace more developed technologies. In particular, integration with in vitro and *in cellulo* libraries will provide high-throughput applications for cyclic peptides screening. A combination of one of these tools with another method or a brand-new platform will likely be considered in order to provide better synthetic and screen machinery [3]. Specifically, these in vitro and in vivo screening libraries enable ultra-high throughput screening accessible to laboratory settings without special resources. The rapid development of computational tools, including *in silico*-guided peptide library generation, will further accelerate cyclic peptide-based drug development. Two-hybrid screening allows for activity-based screening which could be integrated with a well-known library. These features could be increasingly applied to bicyclic peptides to explore versatile modalities and chemical spaces and adapt to novel chemical approaches. Inspired by biosynthetic logic for future directions, expanding the toolkit of current methodologies will broaden the opportunities for structural diversification and enable library integration and functional screening at a high-throughput level. Great structural diversity and broad exploration spaces are guaranteed using versatile means such as ribosomal vs. non-ribosomal and chemical vs. enzymatic platforms. Diverse utilities such as probes for specific proteins or metabolites in vivo will be explored with regard to the selective and strong binding affinity of cyclic peptides. Furthermore, progress in the cyclization of peptides and their screening platforms will enable broader future applications in a variety of fields, including structure diversity and space exploration, therapeutics, drugging of undruggable targets, and building blocks for macromolecules.

## 4. Concluding Remarks

In summary, the biosynthesis of macrocyclic peptides has substantially developed to provide a competitive platform technology in the field of drug discovery, accompanying in vitro display and DNA-encoded library technologies. In contrast to organic synthesis, biosynthesis provides a unique and robust process to identify early-hit cyclic peptides that can be further optimized. Six main approaches of ribosomal and non-ribosomal synthesis (FIT, RaPID, SICLOPPS, RiPPs, NRPS, and chemo-enzymatic synthesis) have been envisioned to extend the applicability of peptide cyclization for the discovery of more advanced peptides with higher affinity. The integration of cyclic peptide libraries was emphasized based on different categories such as mRNA display and phage display. The key features and advantages of each methodology were summarized. The biosynthetic characteristics of in vitro display such as phage display enable structural diversity for natural product-like bicyclic peptides. Bicyclic peptide platforms are shining stars as building blocks for heterotrimeric bicyclic peptide conjugates with linkers to address undruggable challenges. They represent a novel platform for assembling tandems, trimers, and tetramers and drug conjugate via phage screening. More importantly, a variety of biosynthetic perspectives and insights inspired from the novel cyclic peptide technologies were discussed to provide insights into future trends.

## Data Availability

Not Applicable.
